# The Role of Different TET Proteins in Cytosine Demethylation Revealed by Mathematical Modeling

**DOI:** 10.3390/epigenomes8020018

**Published:** 2024-05-02

**Authors:** Karolina Kurasz, Joanna Rzeszowska-Wolny, Ryszard Oliński, Marek Foksiński, Krzysztof Fujarewicz

**Affiliations:** 1 Silesian University of Technology, Akademicka 16, 44-100 Gliwice, Poland; 2 Department of Clinical Biochemistry, Faculty of Pharmacy, Collegium Medicum in Bydgoszcz, Nicolaus Copernicus University in Toruń, Karlowicza 24, 85-092 Bydgoszcz, Poland

**Keywords:** cytosine demethylation, TET proteins, mathematical modeling

## Abstract

In living cells, some reactions can be conducted by more than one enzyme and sometimes it is difficult to establish which enzyme is responsible. Such is the case with proteins from the TET family, capable of converting 5-methyl-2’-deoxycytidine (5-mdC) in DNA to 5-(hydroxymethyl)-2’-deoxycytidine (5-hmdC) and further to 5-formyl-2’-deoxycytidine (5-fdC) and 5-carboxy-2’-deoxycytidine (5-cadC). The estimation of the efficiency of particular TETs in particular oxidative reactions and different cell types is important but experimentally difficult. Here, we propose an approach with mathematical modeling in which methylation and known deoxycytidine modification pathways are presented by 343 possible model versions with assumed different combinations of TET1, 2, and 3 activities in different pathways. Model parameters were calculated on the basis of 5-mdC, 5-hmdC, 5-fdC, 5-cadC, and 5-hmdU levels experimentally assessed in five human cultured cell lines and previously published. Selection of the model versions that give in simulations the best average fit to experimental data suggested that not all TET proteins participate in all modification reactions and that TET3 activity may be especially important in the reaction of 5-fdC removal.

## 1. Introduction

Regulation of gene expression is an extremely complicated process, which relies on different factors, one of which is methylation of cytidine in DNA. The product of methylation is 5-methyl-2-deoxycytidine (5-mdC), sometimes called the fifth nucleotide. Accumulation of 5-mdC in gene promoters leads to inhibition of their expression [1] and is a major epigenetic factor that plays the main role in differentiation during development. In some cancers such as leukemias, groups of genes containing CpG islands in their promoters become hypermethylated and silenced, whereas others can be demethylated and activated [1]. The methylation process is conducted mainly by DNA methyltransferases, DNMT1 in the replication process (genomic imprinting), or DNMT3A/B in de novo DNA methylation [1]. Less is known about the demethylation and enzymes participating in this process. Active demethylation of 5-methyl-2-deoxycytidine (5-mdC) moieties in DNA occurs by oxidation to 5-hydroxymethyl-2-deoxycytidine (5-hmdC) and further oxidation to 5-formyl-2-deoxycytidine (5-fdC) and 5-carboxyl-2-deoxycytidine (5-cadC), and is performed by enzymes of the Ten-Eleven Translocation family (TETs 1, 2, and 3) [2,3], but the particular role of each enzyme from the TET family in each step is not known. Deoxycyditines can also be deaminated and transformed to deoxyuridine by Activation-Induced Cytidine Deaminase (AID) [3,4]. All these modified cytidine forms except 5-mdC are recognized by DNA repair systems and converted back to deoxycytidine in DNA [3].

**Figure 1:**
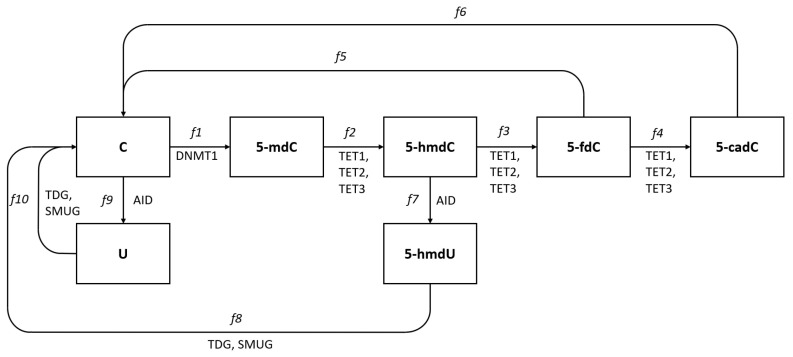
Pathways of deoxycytidine modifications in eukaryotic DNA. Cytidine in DNA is converted to 5-methyl deoxycytidine (5-mdC) by methyl transferase DNMT1, and 5-mdC is converted to 5-hydroxymethylcytosine (5-hmdC) and further to 5-formylcytosine (5-fdC) and 5-carboxylcytosine (5-cadC) by enzymes from the TET family, TET1, TET2, and TET3. Deoxycyditine, 5-mdC and 5-hmdC can also be deaminated and become uracil, 5-mU and 5-hmdU and these reactions are conducted by the AID enzyme. All these modified cytidine forms except the methylated are recognized by the DNA repair systems TDG or SMUG glycosylases and converted back to cytidine in DNA.

We summarize known pathways and enzymes participating in the formation of different deoxycytidine modifications. Pathways leading back to deoxycytidine are simplified in our scheme as TDG or SMUG enzymes that participate only in the first step of DNA repair pathways and in these cases, we do not show the further steps. This is justified as our point of interest is to look for mechanisms leading to modified deoxycytidine forms and their levels. Oxidation reactions of 5-mdC by hydroxylation and further oxidations to 5-fdC and 5-cadC may be conducted by any of the TET1, TET2, or TET3 enzymes [5]. Each of the TETs (1, 2, or 3) may have slightly different affinity to different substrates, and their efficiencies in these reactions may differ. The level of 5-mdC will thus depend on cytidine methylation efficiency and efficiency of 5-mdC conversion to 5-hmdC. These efficiencies are marked as $f_{1}$ and $f_{2}$ on Fig Figure 1 and the rate of changes of the 5-mdC level may be expressed as the difference between $f_{2}$ and $f_{1}$ (production and exchange to another form). Similar expressions can be used for further reactions and the difference between $f_{2}$ and the sum of $f_{3}$ and $f_{7}$ will give the rate of change in 5-hmdC level and differences between $f_{3}$ and the sum of $f_{4}$ and $f_{5}$, the rate of change in 5-fdC level, etc. Experimental studies allow the levels of modified cytidines in DNA to be assessed and in our previous work, we assayed and compared the levels of all modified types of deoxycytidines in the DNA of five human cell lines, HCT116, K562, Me45, Raji, and NHDF [6]. We demonstrated substantial differences in 5-hmdC, 5-fdC, and 5-cadC levels among different cultured cell lines. In the same cells, the levels of transcripts of TET and other enzymes shown in Figure 1 were assessed by the RT-qPCR method [7]. A mathematical modeling approach was recently published and has been extensively applied to DNA methylation and has provided useful insights on the regulatory mechanisms of this process [8]. The goal of this study was, using this experimental data, to propose a mathematical model of methylation and demethylation of cytosine forms, which would be able to predict the levels of different cytosine forms based only on knowledge of enzyme transcript levels and the selection of model structure, which would allow the TET proteins that work on successive stages of 5-mdC transformation to be identified.

## 2. Materials and Methods

The data that we used in our work were experimentally obtained and are described in the article [6]. The data set included levels of different cytidine modifications and levels of participating enzymes DNMT1, TET1, TET2, TET3, AID, SMUG, and TDG.

### 2.1. Cell Lines

Five different cell lines were used in the biological experiments—four cancer lines and one normal line. HCT 116 is an epithelial cell line of colorectal carcinoma, K562 is a lymphoblast cell line from bone marrow from chronic myelogenous leukemia, and Raji is a B lymphocyte cell line from Burkitt’s lymphoma and this cell line contains a Herpesvirus (EBV). These three cell lines were obtained from ATCC. Me45 is a melanoma cell line obtained from the Gliwice branch of the Center of Oncology Maria Sklodowska–Curie Memorial Institute; it was extracted from the cheek skin of an oncology patient. NHDF is the only normal cell line and was obtained from Lonza; it was extracted from the dermis of normal human neonatal foreskin.

### 2.2. PCR Assay

Table 1 presents the average level of expression of enzymes that was assessed by the RT-qPCR method and the level of modification of nucleotides that was determined by the 2D-UPLC-MS/MS method [7].

### 2.3. Mathematical Model

The model includes the pathways shown in Figure 1. We assume that unmodified and modified deoxycytidines create subgroups (compartments) and that in each living cell, there exists “flow” from one subgroup to another. We assume that in particular physiological conditions, the flow between subgroups is equilibrated and gives quasi-stable levels of different modifications which are characteristic of cell types and can be estimated by experimental methods. The flow between “modified deoxycytidine compartments” depends on the presence and activity of enzymes participating in modifications. The efficiency of modification processes and the levels of the modified base could be described, as in chemical reactions, by the product of available substrate levels (“concentrations”) and some proportionality coefficients, and for the reaction in which 5-mdC arises from unmodified dC, it can be expressed as:

(1) $$\begin{array}{c} k_{DMNT1}\cdot C\cdot DNMT1 \end{array}$$

where *C* represents the cytidine “concentration”—the amount of available for methylation deoxycytidine moieties, DNMT1 the “concentration” of DNMT1 enzyme, and $k_{DNMT1}$ is a proportionality coefficient that reflects the affinity of DNMT1 to its substrate. Expression (1) gives the estimation of 5-mdC production rate and is marked in Figure 1 as $f_{1}$. 5-methyl-deoxycytidine is in cells further modified to 5-hmdC by TET enzymes and by analogy, the rate of conversion of 5-mdC to 5-hmdC caused by the TET1 enzyme can be described as

(2) $$\begin{array}{c} k_{1_{TET1}}\cdot mdC\cdot TET1 \end{array}$$

However, there are three TET enzymes that seem able to carry out the conversion of 5-mdC to 5-hmdC and because of this, the rate of 5-hmdC appearance, marked in (Figure 1) as $f_{2}$, can be described by the sum of actions of TET1, TET2, and TET3, each of which can show different affinities to the substrate, and expressed as

(3) $$\begin{array}{c} f_{2}=k_{1_{TET1}}\cdot mdC\cdot TET1+k_{1_{TET2}}\cdot mdC\cdot TET2+k_{1_{TET3}}\cdot mdC\cdot TET3 \end{array}$$

where *TET*1, *TET*2, and *TET*3 are the levels (“concentrations”) of the enzymes and $k_{1_{TET1}}$, $k_{1_{TET2}}$, and $k_{1_{TET3}}$ are the proportionality coefficients reflecting the affinity of each enzyme to 5-mdC substrate, respectively. The change in the level of 5-mdC with time will depend on the rate of its creation and rate of conversion to 5-hmdC and can be described by the differential equation

(4) $$\begin{array}{c} \frac{dmdC}{dt}=f_{1}-f_{2} \end{array}$$

where

(5) $$\begin{array}{c} f_{1}=k_{DMNT1}\cdot C\cdot DNMT1 \end{array}$$

(6) $$\begin{array}{c} f_{2}=k_{1_{TET1}}\cdot mdC\cdot TET1+k_{1_{TET2}}\cdot mdC\cdot TET2+k_{1_{TET3}}\cdot mdC\cdot TET3 \end{array}$$

By analogy, changes in the levels of all other forms with time can be described the same way as differences in their creation and conversion to the other form of 5-mdC. 

5-hydroxymethylcytosine rate of change:

(7) $$\begin{array}{c} \frac{dhmdC}{dt}=f_{2}-f_{3}-f_{7} \end{array}$$

where

(8) $$\begin{array}{c} f_{3}=k_{2_{TET1}}\cdot hmdC\cdot TET1+k_{2_{TET2}}\cdot hmdC\cdot TET2+k_{2_{TET3}}\cdot hmdC\cdot TET3 \end{array}$$

(9) $$\begin{array}{c} f_{7}=k_{AID}\cdot hmdC\cdot AID \end{array}$$

5-formylcytosine rate of change:

(10) $$\begin{array}{c} \frac{dfdC}{dt}=f_{3}-f_{4}-f_{5} \end{array}$$

where

(11) $$\begin{array}{c} f_{4}=k_{3_{TET1}}\cdot fdC\cdot TET1+k_{3_{TET2}}\cdot fdC\cdot TET2+k_{3_{TET3}}\cdot fdC\cdot TET3 \end{array}$$

(12) $$\begin{array}{c} f_{5}=k_{4}\cdot fdC \end{array}$$

5-carboxylcytosine rate of change:

(13) $$\begin{array}{c} \frac{dcadC}{dt}=f_{4}-f_{6} \end{array}$$

where

(14) $$\begin{array}{c} f_{6}=k_{5}\cdot cadC \end{array}$$

5-hydroxymethyluracil rate of change:

(15) $$\begin{array}{c} \frac{dhmdU}{dt}=f_{7}-f_{8} \end{array}$$

where

(16) $$\begin{array}{c} f_{8}=k_{SMUG}\cdot hmdU\cdot SMUG \end{array}$$

Uracil rate of changes:

(17) $$\begin{array}{c} \frac{dU}{dt}=f_{9}-f_{10} \end{array}$$

where

(18) $$\begin{array}{c} f_{9}=k_{TDG}\cdot U\cdot TDG \end{array}$$

(19) $$\begin{array}{c} f_{10}=k_{AID}\cdot C\cdot AID \end{array}$$

The model describing changes in the levels of different cytidine forms in DNA thus contains six differential equations based on the balance between amounts of the different deoxycytidine forms presented in Figure 1. Different parameters for the same enzyme acting in different reactions were assumed (for example for the TET1 enzyme, parameters $k_{1_{TET1}}$, $k_{2_{TET1}}$, and $k_{3_{TET1}}$ for reactions with 5-mdC, 5-hmdC, and 5-fdC, respectively), as the affinity of the same enzyme to different substrates can be different. This approach increases the number of parameters. Conversely, we assume that in the same reaction but in different cell types, enzymes act with the same affinities for their substrates and, for example, parameter $k_{1_{TET1}}$ characterizing the action of TET1 in the oxidation of 5-mdC will be the same in different cell types. Moreover, the equations are linear with respect to the levels of the different forms of cytosine, which is a result of an assumption that the system operates in the linear part of the characteristics even when they are non-linear.

### 2.4. Steady State Solution

It seems reasonable to assume that in cells that express different TETs and other cells participating in cytidine modification enzymes, the modification processes are permanently ongoing and some dC become methylated and further converted to the next forms. The assayed levels of each form in DNA reflect the equilibrium between the efficiency of enzymes catalyzing the conversion of one form to another and the whole process can be treated as equilibrated flow from one form to another starting from dC and finishing as dC (Figure 1). The levels of different cytidine forms assayed in different cell types seem to fluctuate around some characteristic values for each cell type and in some approximation can be assumed as quasi-constant giving $\frac{dmdC}{dt}$, $\frac{dhmdC}{dt}$, $\frac{dfdC}{dt}$, $\frac{dcadC}{dt}$, $\frac{dU}{dt}$, $\frac{dhmdU}{dt}$ equal to zero. In such conditions, the rate of creation of some cytidine form must be equal to its rearrangement to the other forms, and the flows shown in Figure 1 can be described by the following equations: $f_{1}=f_{2}$, $f_{2}=f_{3}+f_{7}$, $f_{3}=f_{4}+f_{5}$, $f_{4}=f_{6}$, $f_{7}=f_{8}$, $f_{9}=f_{10}$.

From the point of view of state variables (levels of various forms of cytosine), the model is linear. The stability of the steady-state solution has been checked. For all positive parameters and protein levels, the real parts of all eigenvalues of the system matrix are negative, which confirms the stability of the equilibrium. Moreover, because all eigenvalues are real, the time solution of the system is aperiodic.

### 2.5. Estimation of Parameters Characterizing the Action of Enzymes

To be able to calculate the real values of flows between groups of modified cytidines, one needs to know the values of parameters characterizing the action of participating enzymes and the levels of modified cytidines in each group. Assuming that parameters describe the affinities of enzymes to specific substrates and that these affinities are the same in different cell types, to calculate their values, one could use the equations comparing flows in steady-state conditions together with known levels of different cytidine modifications and the levels of participating enzymes DNMT1, TET1, 2, and 3, AID, SMUG, and TDG. In our previous work, we experimentally assessed the levels of 5-mdC, 5-hmdC, 5-fdC, and 5-cadC in five human cultured cell lines and in the same lines, estimated the levels of the above-mentioned enzymes on the basis of their transcript levels [6]. These data allowed us to estimate model parameters with the assumption that all TET enzymes are active in all the reactions shown in Figure 1. To calculate the parameters of the model, we used the non-negative linear least squares (NNLS) method, which computes a non-negative solution to a linear least squares problem [9]. NLLS is a constrained version of the least squares problem in which the coefficients are not allowed to become negative.

Using such an assumption and with estimated parameters, we performed a model simulation and calculated the performance index showing how well values obtained from the simulation fit the real experimental data that were used for parameter estimation. The value of the mean square error performance index was $1.2036\times 10^{-5}$. However, one should remember that this index may be optimistically biased because of the risk of “over-training”, which is particularly strong for models with a large number of parameters and this is the case with our model.

To obtain a more reliable model evaluation we used a cross-validation method (the quality of the model was assessed on the data not used for parameter estimation—details are given in the following sections). The performance index obtained by cross-validation was significantly worse, suggesting that the model does not reflect the real situation. The problem may lie in the fact that some TET enzymes may not interact with all substrates taken into account in the model. The TET1, TET2, and TET3 enzymes are able to oxidize different forms of cytosine and work together. However, not all TET enzymes need to participate in each cytosine transformation (demethylation), which would correspond to possible differences in the mathematical models (with a lack of some TETs in some reactions). Even though we have the experimentally assessed cytidine modification levels and enzyme levels, we are still not able to tell what roles particular TET enzymes play in particular reactions (flows between compartments) and consequently, how to use the model for previewing the levels of cytidine modifications in cells with unknown modification levels.

### 2.6. Model Selection

To find out the role of TETs in different flows, we created a series of models with all possible combinations of TET1, 2, and 3 actions in different reactions of cytidine modification. There are 343 possible versions of such models and several examples are shown in Table 2. To choose the model structure containing the combination of TETs that best fit to the experimental data, we calculated model parameters for each of the 343 model structures with a cross-validation method in which the results of experiments with five cell lines were taken into account and parameters of some TETs in some reactions in specific model versions could be equal to zero (i.e., an assumption that that particular TET does not interact with that particular substrate).

### 2.7. Performance Index

In the cross-validation method applied to the results of experiments with five cell lines for each model structure, five performance indexes were obtained in the form of the parameters estimated on the basis of experimental results obtained for four cell lines with the removal of the fifth cell line; next, for each of these parameter sets, model simulations previewing the levels of modified cytidines were carried out and compared with experimental assessments in the removed cell line. Finally, for each of the 343 model structures, the overall quadratic performance index measuring its predictive ability was defined as follows:

(20) $$\begin{array}{c} J_{CV}=\frac{1}{2}\sum_{m=1}^{M}\sum_{n=1}^{N}{\left(x_{n}^{m}-d_{n}^{m}\right)}^{2} \end{array}$$

where $x_{n}^{m}$ is the prediction of the level of the *n*-th form of cytosine in the *m*-th removed cell line and $d_{n}^{m}$ is the corresponding measurement. For our experimental data set, we have M=5 (number of cell lines) and N=6 (number of equations). The cross-validation performance index (20) can be understood as a generalization measure of a particular model variant.

We used the static model (for the steady-state solution of differential equations) and the resulting linear (with respect to the parameters) model to find its optimal structure. This is a routine approach, for example, in statistics, where models are usually static. All 343 possible combinations of parameters $k_{1_{TET1}},k_{1_{TET2}},k_{1_{TET3}},k_{2_{TET1}},k_{2_{TET2}},k_{2_{TET3}},k_{3_{TET1}},k_{3_{TET2}},\;and\;k_{3_{TET3}}$ were analyzed. For each structure, the cross-validation performance index (20) was calculated—see Table 3, in which the 10 best results are presented alongside the full structure and the worst structure with the highest performance index.

## 3. Results and Discussion

### 3.1. Biological Findings Resulting from Model Selection

The aim of this work was to characterize the role of the TET enzymes participating at different stages of the process of DNA demethylation and to find out if different TETs are used similarly by different cell types.

**Figure 2:**
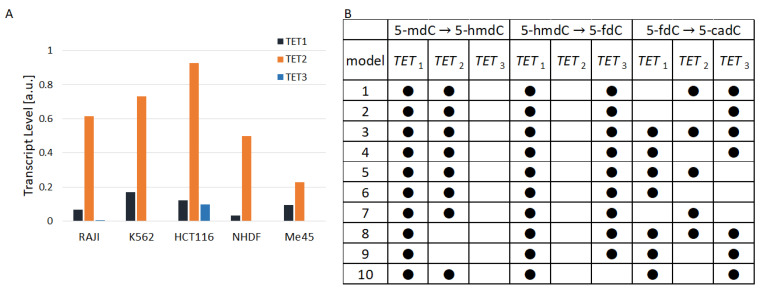
(**A**): The TET levels in the studied cell types (**B**): hypothetical participation of TET enzymes in the 10 models (with lowest performance index) that best fit to the experimental data.

TET1, TET2, and TET3 are differently expressed in the same and different cell types Figure 2A. In all cell types that we studied, except Me45, TET2 had a much higher level than TET1. The ratio of TET2 to TET1 raned from 1 in Me45 cells to 15 in Raji cells and the ratio of TET2 to TET3 ranged from a value of 8 in Me45 cells to nearly 190 in Raji cells. Figure 2A shows the proportions of TET1, TET2, and TET3 transcripts in the studied cell lines calculated as average values from transcript assays performed at different time points. Figure 2B shows 10 examples of the structure of models assuming the lack of activity of different TETs in particular reactions. The models in the table shown in Figure 2B are ordered on the basis of their performance index (best similarity to experimental results). Black dots in the table indicate active TET enzymes in cytidine oxidation reactions from the scheme presented in Figure 1. The best result (fitting to experimental results) was obtained for the case in which between 5-mC and 5-hmdC, TET1 and TET2 are active, while between 5-hmdC and 5-fdC and also between 5-fdC and 5-cadC, TET1 and TET3 are active. Nevertheless, the performance index J of the best model is not much different from J of the second-best and subsequent models. Instead of looking at only one model structure with the lowest performance index, one can take into account 10 successive model structures with the lowest J. Analyzing these 10 best structures one could conclude that in steady states when cytidine modifications are kept at the level most similar to that found experimentally:

- During the transformation 5-mdC → 5-hmdC, there is no enzymatic activity of TET3;
- During the transformation 5-hmdC → 5-fdC, there is no enzymatic activity of TET2;
- During the transformation 5-fdC → 5-cadC, TET1 can be replaced by TET3 or TET2.

None of these findings are directly confirmed by recent publications. In some publications, one can find data that suggest that in the reaction of 5-mdC conversion into 5-hmdC, the TET3 enzyme plays some role; for example, a decrease in 5-hmdC was observed after the knockdown of the TET3 gene [10]. The observation that TET3 may be dispensable in the conversion of 5-mdC to 5-hmdC shown by our models with the best structure may be the result of differences between the levels of TET1, TET2, and TET3 and competition between them.

### 3.2. Comparison of Model Simulations in Respect to Specific Modifications

**Figure 3:**
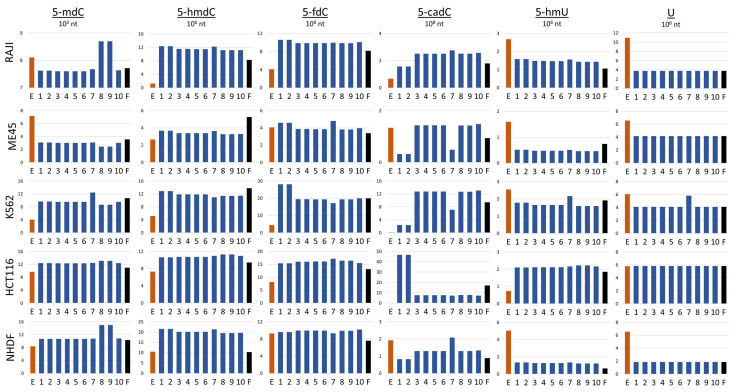
Comparison of the estimations of cytosine modifications obtained by experimental methods (red bars) and mathematical model simulations (blue and black bars) in different cell lines, E; experimental assay, 1–10; models with the best performance indexes, F; model with all TET enzyme types active in all reactions.

According to our model simulations, the best fit between predicted and experimentally assessed cytidine modification levels can be obtained when we exclude TET3 from the reaction between 5-mdC and 5-hmdC and TET2 from the reaction between 5-hmdC and 5-fdC. In the transformation of 5-fdC to 5-cadC, the results of simulations indicate that to obtain a good fit between model simulation and experiment, TET1 would be indispensable and TET2 and TET3 could exchange. The worst result (highest performance factor) was obtained when TET1 was excluded from the first two reactions 5-mdC to 5-hmdC and 5-hmdC to 5-fdC Table 3. However, the performance index by definition compares the level of all cytidine modifications and in some cases, a high level in one type of modification may be compensated by a low level somewhere else. In the next step, we compared the levels of modifications obtained by the simulation performed by models with structures presented in Figure 3. To achieve this, we assessed the general ability of a particular model to preview the levels of cytidine modifications. Next, we analyzed how different combinations of TETs assumed in the models with the best performance index may predict the levels of particular cytidine modifications in different cell lines. The levels of modified cytosine predicted by the best simulation models with the lowest performance index for each type of cytosine modification are shown in Figure 3. Each row in the Figure shows the results obtained for a different cell line (Raji, K562, HCT116, Me45, and NHDF). In the graphs, the results of experimental assays are shown with red bars, those calculated with the help of the optimal models with blue bars, and those predicted by the model with all TETs active with black bars.

Looking at the results obtained with the 10 models that best fit to the experimental data, one can see that all these models predict similarly the levels of different cytidine modifications in most of the cell lines (Figure 3). However, some models differ from others in predicting particular modifications and are cell-type-specific—see models 8 and 9, which preview higher levels of 5-mdC but only in Raji and NHDF cells. Models 1 and 2 also are specific in respect to some modifications and cell types. They predict higher levels of 5-fdC in K562 and lower levels of 5-cadC in Raji, Me45, and NHDF cells, However, simulations by these models predict higher levels of 5-cadC in HCT116 cells. Some modification and cell type specificity can be seen also in the simulations by model 7, in which some cell types predicted slightly higher levels of 5-fdC and lower levels of 5-cadC in Me45 and K562 cells but higher levels of 5-cadC in NHDF cells. The simulations of model 7 for K562 also showed higher levels of dU and 5-hmdU than other models. None of the model predictions reflected well all modification levels assessed in the experiments, however, the model simulations may suggest the role of TETs in different pathways of modification and differences in these pathways between cell types. In models 8 and 9, TET2 is inactive in 5-mdC hydroxylation. In Raji cells, this enzyme was most efficiently expressed (Figure 2A), and the lack of its engagement in the first step of demethylation may cause hte accumulation of more 5-mdC. Conversely, however, models with TET1 and TET2 active in this reaction predict underestimated levels of 5-mdC. Clearly, differences in different modification levels observed in different cell types for the same model can be the result of differences in the levels of enzymes participating in demethylation in different cell types. For model construction and simulations, we did not employ real concentrations of particular enzymes participating in the reactions but instead, approximated protein levels from the levels of their transcripts, which are easier to obtain. Transcript and protein levels are not always proportional; in some cases, translation can be inhibited without mRNA degradation [11]. In the scheme for cytidine demethylation presented in Figure 1, we show that 5-hmdC may be further oxidated with some of TETs to 5-fdC or deaminated to 5-hmU and the conversion of 5-fdC and 5-cadC to *C* may proceed through base excision repair based on different glycosylases in different cells. In fact, the uracil present could be removed by four different proteins (TDG, UNG, SMUG1, and MBD4) [12], although these were not all taken into account in the modeling and the performed experiments. The differences in levels of cytosine modifications could be also caused by the lack of information on the thymine, in that this may be produced by deamination of 5-mdC and was not taken into account in our model. In future work, we plan to incorporate the above-mentioned mechanisms into our model. Additionally, the levels of 5-hmdU and 5-hmdC strongly depend on oxidative conditions, i.e., the presence of vitamin C enhances significantly the level of 5-hmdU in human cells [7]. Nevertheless, it seems that the type of modeling proposed here, and especially, the way of fitting the models to some experimental data, may provide us with a tool for studies of not only the action of TET enzymes but also other modifications and cellular reactions.

Model simulations show that by inactivating one of the TET enzymes in one particular reaction (for example, by specific modification of protein participants in the complex), it is possible to change the level of a single modification while maintaining the levels of other modifications in the cell. 5-hmdC and 5-fdC are like 5-hmdU and 5-mdC in terms of the epigenetic marks that may play a specific role in modulating gene expression [13]. The existence of specific roles requires mechanisms that allow for specific modulations of the action of the TET enzymes. In spite of the fact that none of our models predicted results that reflected well all modification levels assessed in the experiments, the model simulations may suggest the ways in which epigenetic marks are modulated and the role of TETs in these pathways.

## 4. Conclusions

The article proposes an original mathematical model of cytosine methylation and demethylation. Experimental data for five cell lines were used to create the model. Instead of building five different models for each cell line, we assumed that similar cytosine transformation mechanisms govern all the cell lines and that differences in the levels of different forms of cytosine are due to different enzyme levels. In particular, we focused on the role of different forms of the TET enzyme at different stages of cytosine transformation. Using one common data set to estimate model parameters allowed us to examine which of the 343 possible model structures has the best generalization ability. The analysis of the best structures allowed us to answer the question about the probable participation of different forms of the TET enzyme at different stages of cytosine transformation. Some of our conclusions have been confirmed in the available literature, some are new.

The model in its current form is only suitable for describing the epigenetic transformation of cytosine, but the way it was built and the parameter estimation method are universal and can be applied to other cellular or, more broadly speaking, biological processes.

The model, like any mathematical model, is a simplified model in which some phenomena are taken into account and some are not. In our further work, we plan to expand the model to take into account other phenomena, for example, the impact of stressful factors, such as ionizing radiation, for which we also have experimental data.

## Figures and Tables

**Table 1:** Levels of transcripts of the AID, SMUG1, TDG, DNMT1, and TETs genes in various cell lines.

	RAJI	K562	HCT	NHDF	Me45
AID	0.009824	0.010643	0.086131	0.002238	0.001404
SMUG1	1.195942	1.19889	16.01112	0.914257	0.361182
TDG	0.29466	0.174258	0.449975	0.030231	0.076355
DNMT1	0.092437	0.236583	0.081149	0.082171	0.038231
TET1	0.064967	0.169337	0.056229	0.033814	0.096231
TET2	0.614074	0.73099	0.620413	0.497276	0.043056
TET3	0.004811	0.003187	0.027295	0.002800	0.000876

**Table 2:** Examples of possible combinations of TET1, 2, and 3 actions in different cytidine modification reactions.

5-mdC → 5-hmdC			5-hmdC → 5-fdC			5-fdC → 5-cadC		
$TET_{1}$	$TET_{2}$	$TET_{3}$	$TET_{1}$	$TET_{2}$	$TET_{3}$	$TET_{1}$	$TET_{2}$	$TET_{3}$
•	•	•	•	•	•	•	•	•
	•	•	•	•	•	•	•	•
•	•	•		•	•	•	•	•
•	•	•	•	•	•		•	•
⋮	⋮	⋮	⋮	⋮	⋮	⋮	⋮	⋮
•	•		•	•		•	•	
⋮	⋮	⋮	⋮	⋮	⋮	⋮	⋮	⋮
•			•			•		

**Table 3:** Overall quadratic performance indexes for different structures; black dots • mean that a particular parameter is enforced to work. The 10 best-performing structures are presented, as well as the full structure and the worst structure with the highest performance index.

	5-mdC → 5-hmdC			5-hmdC → 5-fdC			5-fdC → 5-cadC		
**Performance Index**	$TET_{1}$	$TET_{2}$	$TET_{3}$	$TET_{1}$	$TET_{2}$	$TET_{3}$	$TET_{1}$	$TET_{2}$	$TET_{3}$
$1.2036\times 10^{-5}$	•	•		•		•		•	•
$1.2043\times 10^{-5}$	•	•		•		•			•
$1.2101\times 10^{-5}$	•	•		•		•	•	•	•
$1.2101\times 10^{-5}$	•	•		•		•	•		•
$1.2511\times 10^{-5}$	•	•		•		•	•	•	
$1.2511\times 10^{-5}$	•	•		•		•	•		
$1.2687\times 10^{-5}$	•	•		•		•		•	
$1.3110\times 10^{-5}$	•			•		•	•	•	•
$1.3110\times 10^{-5}$	•			•		•	•		•
$1.3116\times 10^{-5}$	•	•		•			•	•	•
⋮	⋮	⋮	⋮	⋮	⋮	⋮	⋮	⋮	⋮
$3.6277\times 10^{-5}$	•	•	•	•	•	•	•	•	•
⋮	⋮	⋮	⋮	⋮	⋮	⋮	⋮	⋮	⋮
0.0167			•		•		•		

## Data Availability

The data used in the study are included in the article, further inquiries can be directed to the corresponding author.

## Abbreviations

The following abbreviations are used in this manuscript:

TET	ten-eleven translocation enzyme (concentration)
5-mdC	5-methyl-2’-deoxycytidine
5-hmdC	5-(hydroxymethyl)-2’-deoxycytidine
5-fdC	5-formyl-2’-deoxycytidin
5-cadC	5-carboxy-2’-deoxycytidine
5-hdmU	5-Hydroxymethyluracil
*U*	uracil (concentration)
